# Mixed Matrix Poly(Vinyl Alcohol)-Copper Nanofibrous Anti-Microbial Air-Microfilters

**DOI:** 10.3390/membranes9070087

**Published:** 2019-07-17

**Authors:** Elise des Ligneris, Ludovic F. Dumée, Riyadh Al-Attabi, Erwan Castanet, Jürg Schütz, Lingxue Kong

**Affiliations:** 1 Deakin University, Geelong, Institute for Frontier Materials, Waurn Ponds 3216, Victoria, Australia; 2 Commonwealth Scientific and Industrial Research Organization CSIRO, Waurn Ponds 3008, Victoria, Australia

**Keywords:** sol–gel electrospinning, anti-bacterial activity, air filtration, composite nanofibers

## Abstract

Membranes decorated with biocide materials have shown great potential for air sanitization but can suffer from biocide agent leaching by dissolution in water. In order to tackle the diffusion of biocide metal ions from the fiber matrix, composite nanofiber membranes of poly(vinyl alcohol) (PVA) cross-linked with copper (II) acetate have been successfully engineered via sol–gel electrospinning, providing a stable mean for air bactericidal microfiltration. The novelty lies in the bonding strength and homogeneous distribution of the fiber surface biocide, where biocide metals are incorporated as a sol within a polymer matrix. The electrospinning of bead-free composite nanofibers offered over 99.5% filtration efficiency for PM_2.5_, with a theoretical permeance above 98%. The PVA/copper nanofiber membranes also showed satisfactory anti-bacterial performance against the gram-negative *Escherichia coli* within 24 h, making them promising materials for the remediation of airborne bacteria. The mechanical and chemical stability of the engineered nanocomposite electrospun nanofiber webs added to the natural biodegradability of the materials, by offering ideal low-cost sanitary solutions for the application of air disinfection in both indoor and outdoor fitting a circular economy strategy where advanced materials are redesigned to be sustainable.

## 1. Introduction

Air sanitization plays an important role in the limitation of the spread of infections caused by pathogenic micro-organisms, such as bacteria *Legionella pneumophila* [1]. Anti-bacterial micro-filters are thus a promising material solution as sanitary means such as in public transportation or households, where a large conventional ventilation and air conditioning system cannot be implemented [1,2]. Emphasis is to be put on the environmentally friendly nature and cost-effective fabrication and disposal of the filters to generate materials relevant to a circular economy scheme [3,4]. Conventional air filters typically contain glass fibers mixed with polymers, such as poly(ethylene) and poly(propylene), which are difficult to biodegrade and may release harmful secondary contaminants such as bacteria from the after-use handling [4].

Fibrous filters and in particular electrospun nanofiber filters demonstrated to combine over 95% filtration efficiency for particulate matter over 0.3 μm in size, with a low air flow resistance [5]. In the electrospinning process, a homogeneous polymer precursor is pushed through a positively charged needle, and upon the predominance of the electric force over the precursor surface tension at the needle tip, a fibrous jet is formed and projected toward a grounded collector, resulting from the Coulomb charges attraction/repulsion interactions [6,7]. The electrospinning technique allows for the control of the nanofiber diameter and mechanical strength, as well as for the materials porosity and homogeneity in pore size distribution, contributing to the filter performance [4,6,7].

Considering an electrospun membrane pore size below a few microns and the 0.3 to 5.0 μm particle size range selected in this study, the mechanisms of straining for PM_2–5_, inertial impaction, and interception for PM_0.3–2_ can thus be expected to be predominant [8]. Inertial impaction occurs when the particle inertia hits the fiber even though the air had flowed around the fiber. In the case of a smaller particle size such as PM_0.3_, the force balance is modified resulting in the particle flowing with the air stream, however interception by the fiber can occur as the particle tries to flow around it [8]. This effect is further enhanced at the lower end of this particle size range by Brownian motion, because the random-walk movement caused by thermal energy of the aerosol particle increases the chances for it to be intercepted by the fiber.

In order to ally anti-bacterial activity with separation performance, functionalization of nanofibers post-electrospinning with biocide agents, such as bronopol, thiocyanic acid, or selected metal nanoparticles has been investigated [9,10,11,12,13,14]. However, the use of solvents during functionalization and the potential biocide agent leaching are limitations preventing an industrial-scale application. The inclusion of the biocide agent within the nanofibers, hence in the electrospinning precursor has thus been considered as an alternative to tackle the potential agent leaching issue. Indeed, the coordination of metal atoms present under ionic form with polymer chains imposes a higher reaction energy required to free the metal ions, in comparison with materials where solid metal biocides are deposited or grown at the nanofiber surface as previously reported, thus rate-limiting potential leaching [15]. Among biocides, metal nanoparticles such as silver or copper were among those showing the highest bactericidal potential [9,10,14,16,17,18]. However, the inclusion of a metal salt in an electrospinning precursor will have an impact on the local charge reorganization and conductivity changes within the electrospun jet upon its stretching and during solvent evaporation [19,20,21]. Indeed, a free population of metal ions within the electrospinning precursor will thus increase the precursor conductivity and could incur electrospinning instabilities, depending on the precursor loading, either by an inconstant drawing of solution at the needle tip or upon local charge reorganization when the jet solidifies [19,22]. An immobilization of the metal ion within the precursor will thus downgrade the precursor conductivity, thus allowing for a higher loading in metal ions, while still ensuring a relatively undisturbed electrospinning [19,22]. A way to stabilize the metal center is to synthetize a sol–gel of crosslinked polymer chains with metal ions, which will also limit its ability to leach out during operation [23].

This study reports the fabrication of a highly efficient, sustainable, and green fabricated bactericidal air-microfilter. Copper is a known affordable anti-bacterial agent, and poly(vinyl alcohol) (PVA) is a biodegradable polymer that also showed to be highly compatible with copper salt to form a sol–gel via crosslinking, also increasing its water resistance suitable for air filtration in relatively humid conditions applications [20,24,25]. Copper as a biocide agent and PVA as a green polymer precursor were thus considered to limit the cost of production and the environmental footprint. Nanofiber webs of crosslinked PVA by copper (II) acetate were successfully synthetized by sol–gel electrospinning. The air filtration performance of PVA/copper nanofiber webs was first investigated, highlighting the impact of electrospinning parameters onto the separation performance. Then, the anti-bacterial effect of PVA/copper composite nanofibers was successfully determined against the well-reported gram-negative *Escherichia coli* strain (standard test ISO 20645—adapted). Although being a hydrophilic strain, using *E. coli* enables a comprehensive anti-microbial performance comparison with the literature, and this bacteria presents similarities in size and morphology with airborne gram-negative *Legionella pneumophila*. The here presented development of PVA/copper nanofiber microfilters thus opens a way toward means of green and affordable air sanitization.

## 2. Materials and Methods

### 2.1. Materials and Chemicals

Poly(vinyl alcohol) (PVA) (M_w_ 1,300,000), copper II acetate monohydrate, and glacial acetic acid 99 wt % were purchased from Sigma Aldrich (Milwaukee, WI, USA) and used as received. For the anti-bacterial test, *E. coli* strains (ATCC 8739) were obtained from Thermo-Fischer (Melbourne, Victoria, Australia).

### 2.2. Synthesis of PVA/Copper Nanofibers

#### 2.2.1. Preparation of the PVA/Copper Electrospinning Precursor

In a typical procedure, a 10 wt % aqueous solution of PVA was prepared in a capped beaker and agitated using magnetic stirring at 80 °C for 3 h. An aqueous solution of copper acetate and acetic acid was prepared in a 1:1 weight ratio in a second capped beaker and stirred for 1 h at room temperature. The PVA solution was then slowly incorporated to the copper-containing solution and stirred for at least 6 h at 60 °C, forming a homogeneous sol–gel, and reaching a PVA:Cu balance of 60:40 wt %. Acetic acid was here used as a stabilizer to avoid the hydrolysis of the PVA chains [26]. The PVA/copper precursor was characterized to possess a surface tension around 50 mN/m using pendant drop analysis performed on a contact angle platform with the Attension Theta software. The precursor showed a dynamic viscosity of 1.92 Pa.s, determined using a HR-3 Rheometer (Melbourne, Australia) at ambient temperature. The conductivity of the precursor was determined to be 1.71 mS at 21 °C, using a TPS WP 81 multimeter (Melbourne, Australia) fitted on a stirring plate.

The formation of the sol–gel with the cross-linking of PVA chains by copper atoms, producing water molecules, can be expressed as in the Equations (1,2):

Cu(CH_3_COO)_2_·H_2_O + H_2_O ⇆ Cu(OH)_2_ + 2CH_3_COOH (1)

2 (CH_2_CHOH)_n_ + nCu(OH)_2_ ⇆ 2nH_2_O + (CH_2_CHOCuOCHCH_2_)_n_ (2)

#### 2.2.2. Electrospinning of the PVA/Copper Nanofibers

A KD Scientific syringe pump system was used to deliver the precursor to a 5 mL Terumo syringe fitted with a stainless steel needle connected to a high voltage power supply provided by a Gamma High Voltage Research HV and pointing toward a flat aluminum collector. The electrospinning parameters used in the fabrication of the three PVA/Copper nanofiber webs considered in this study are reported in Table 1.

As different spinning conditions translate in different jet trajectory and acceleration, and thus a different spinning duration for the same web thickness, it is challenging to accurately estimate the appropriate spinning duration for each membrane. However, the membranes thickness was maintained in a similar range, of 115 ± 15 µm through trial and error.

### 2.3. PVA/Copper Nanofiber Web Characterization

#### 2.3.1. Nanofiber Morphology and Diameter

Nanofiber and nanofiber web morphology was assessed using scanning electron microscopy (SEM) on a Zeiss Supra 55VP FEG SEM (Zurich, Switzerland), at 5 keV electron high tension, and 6 mm working distance. The samples were coated with a 5 nm thick gold layer via gold sputtering prior to imaging. Fiber diameter and diameter distribution were assessed via analysis on the SEM images using the G-IMP software (Berkeley, California, USA).

#### 2.3.2. Membrane Pore Size

Membrane bubble point and pore size distributions were characterized using a Quantachrome Instrument Capillary Flow Porometer 3gZh (San Francisco, California, USA). The tests were performed in the wet-up/dry-up mode, using for the wet phase profile as the reference liquid, with a surface tension of 15.9 dynes/cm. The pressure was varied between 0.32 and 2.56 bar to cover the range of material pore size between 250 nm and 2 µm.

Nanofiber pore size and pore volume were not characterized by conventional BJH (Barrett Joyner Halenda) method, as the calculation relies on the shape of the nanofiber pore, which could not be reasonably assumed.

#### 2.3.3. Nanofiber Surface Composition

The verification of the chemical functional groups present at the surface of the PVA/Copper nanofibers before and after air-filtration of KCl microcrystals was performed using attenuated total reflectance Fourier transform infrared (ATR-FTIR) techniques. Spectra were acquired on a Bruker Vetex-70 FTIR spectrometer (Billerica, MA, USA) and recorded in the range of 600–4000 cm^−1^ at a resolution of 4 cm^−1^. A total of 64 spectra were averaged for each measurement.

Further elemental analysis to assess the strength of the copper presence at the composite nanofibers surface was performed via X-ray photospectroscopy (XPS) on a Thermo-Fischer Scientific K-Alpha+ X-ray photoelectron spectrometer (Berkeley, California, USA), with a photon energy of 1253.6 eV, a line width 0.7 eV, and a voltage of 486.7 hv (Mg K-alpha filament). A flood gun was used to avoid charging. Deconvolution of peaks was performed with the CasaXPS software (San Francisco, California, USA).

#### 2.3.4. Membrane Moisture Resistance

Nanofiber web hydrophilicity was assessed post-immersion for 24 h in water (part of the growth solution from the anti-bacterial protocol testing above an agar medium at 37 °C temperature) by SEM image analysis.

### 2.4. Application of PVA/Copper Nanofibers in Anti-Bacterial Air-Filtration

#### 2.4.1. Air Filtration Performance of PVA/Copper Nanofibers

**Figure 1:**
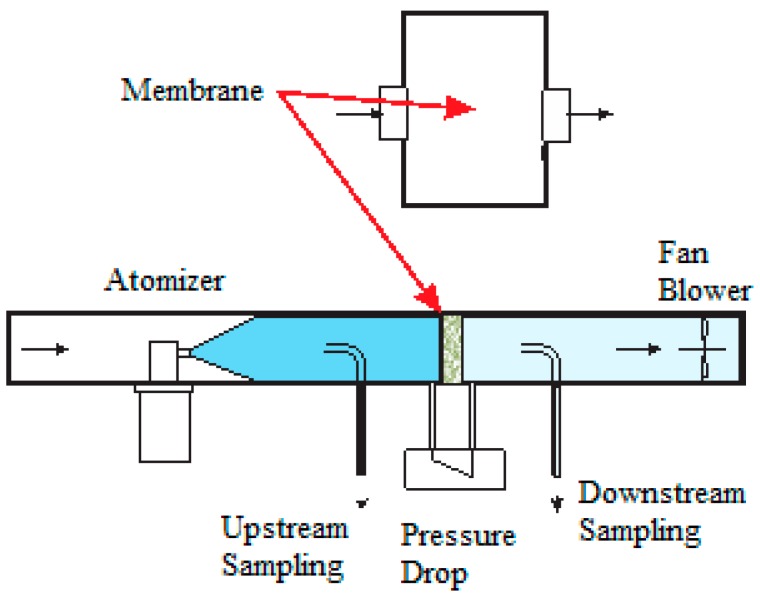
Schematics of the home-made particle counter filter test instrument, Permission to reprint from [27].

A home-made particle counter filter test Instrument PCFTI was used for air separation testing, as presented in Figure 1 [27]. A 20 g/L solution of potassium chloride was used as aerosol by the means of an atomizer to create a broad distribution of particles centered near 600 nm median particle diameter that is analyzed in 6 bins of particle sizes centered at 300, 500, 700 nm, as well as 1.0, 2.0, and 5.0 µm [27]. Two TSI particles counters were used to measure the particles concentration above and under the membrane. A cellulose acetate support was used under all self-standing electrospun membranes. In a typical procedure, a first run without aerosol was carried out to determine a suitable airflow to further use for testing, chosen in function of the membrane pressure drop, and measured with a TSI airflow meter. A minimum of five tests were performed for each membrane, following a protocol adapted from the sodium flame test for air filters standard, under hygrometric conditions below 65% relative humidity (RH).

SEM imaging was performed to image the potential nanofiber delamination after test and perm-porometry tests were carried out to assess the potential membrane pore widening. The membrane quality factor *QF* for a particle size bin *x* was determined following the Equation (3):

(3) $${\mathrm{QF}}_{x}=\frac{-\ln\left(P_{x}\right)}{\Delta p},\;\mathrm{in}\;{\mathrm{kPa}}^{-1}\;\mathrm{or}\;{\mathrm{QF}}_{x}=\frac{-\ln\left(P_{x}\right)}{\Delta p}\;\times \;V_{f}\times \eta ,\;\mathrm{in}\;\mathrm{nm}$$

where $P_{x}$ is the penetration for the particle size bin *x*, Δ*p* the membrane pressure drop, η the viscosity of air, and $V_{f}$ the face velocity. Particle size bins considered are in µm [0.3, 0.5], [0.5, 0.7], [0.7, 1.0], [1.0, 2.0], [2.0, 5.0], and [5.0, 25].

#### 2.4.2. Anti-Bacterial Activity of PVA/Copper Nanofibers

PVA/copper nanofibers anti-bacterial potential was qualitatively assessed using the gram-negative *Escherichia coli* strain, following an adaptation for nanofiber materials of the standard protocol ISO 20645 [28]. The standard ISO 20645 states the protocol for the determination of antibacterial activity for textile fabrics via the agar diffusion plate test. Strains of *E. coli* were grown in a pre-prepared broth from commercial trypton soya broth. Once a concentration of 1–5 × 10^8^ CFU/mL was reached, measured by spectrophotometry from a calibration curve, the broth was inoculated into an agar preparation from commercial trypton soya agar, and poured into 90 mm petri dishes, previously layered with a bacteria-free trypton soya agar. Nanofiber web samples, weighing 1.5 ± 0.02 mg each were placed in the center of a petri dish. The test was repeated twice per sample. Photographs of the inhibition zone were taken after 24 h incubation at 37 °C. Further SEM imaging was performed to assess the impact of the bacteria presence across the nanofiber mats.

## 3. Results and Discussion

Three PVA/copper nanofiber membranes were electrospun in different conditions of electric field and solution volume at the needle tip to help establish a composite nanofiber membrane profile suited for air filtration. The resulting membrane morphology, pore size, and the fiber diameter are presented below and further correlated to the air filter performance, giving an insight of the preferable electrospinning conditions for a polymer/metal composite precursor. Then, the anti-bacterial potential of the PVA/copper nanofibers is showcased.

### 3.1. PVA/Copper Air Microfilters

**Figure 2:**
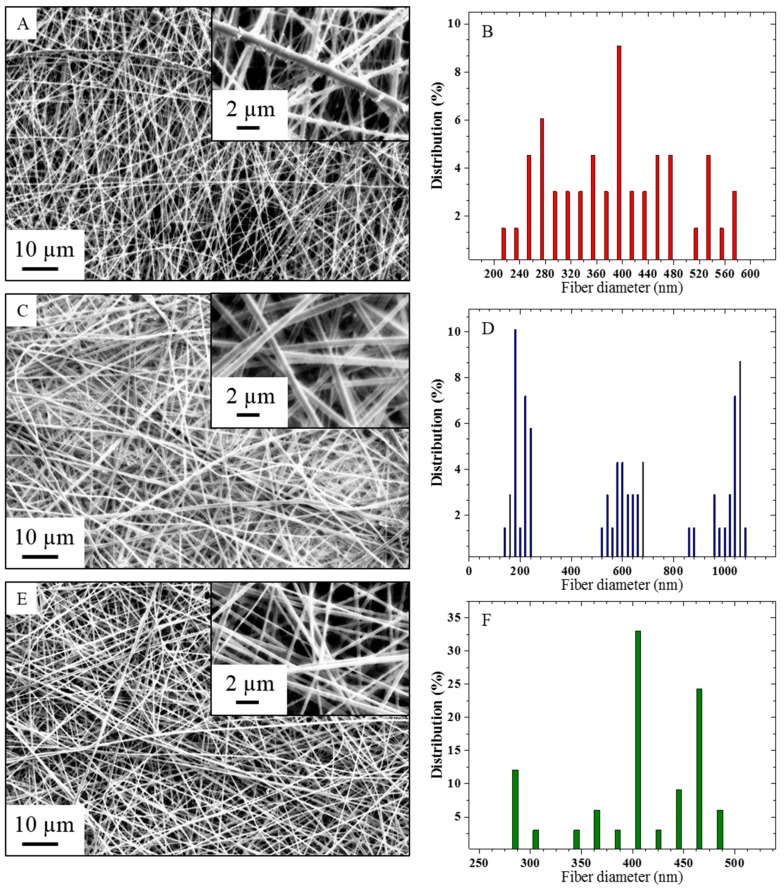
(**a,b**) Scanning electron microscopy (SEM) micrograph and diameter distribution for nanofibers electrospun at 18 kV/0.2 mL/h. (**c,d**) SEM micrograph and diameter distribution for nanofibers electrospun at 16 kV/0.5 mL/h. (**e,f**) SEM micrograph and diameter distribution for nanofibers electrospun at 12 kV/0.6 mL/h.

The morphologies of the three composite nanofiber membranes electrospun in the conditions reported in Table 1 were observed on the SEM micrographs presented in Figure 2, along with their respective nanofiber diameter distribution.

The composite nanofibers presented in Figure 2a,b show a nanoscale roughness with the protrusion of copper-based crystals of above 100 nm in the otherwise relatively smooth electrospun fibers with an average diameter of 318 nm. Electrospinning at 18 kV voltage and 0.2 mL/h feed rate, where values are within the range of usually reported conditions for metal/polymer composite nanofibers, showed secondary jet electrospinning instability as well as the formation of copper cluster, plausibly resulting from an unfavorable electric charge reorganization during stretching of the accelerated jet and a longer precursor polarization duration along the steel needle [22,26,29,30,31]. The relatively high voltage applied onto the conductive precursor also explains the wide distribution of fiber diameters, with a standard deviation of 139 nm. Indeed, studies reported that during electrospinning, the electric field produced by the surface charge, normal to the jet, was slightly larger than the tangential electric field [31,32,33]. As the axi-symmetrical instability is due to the tangential electrical sheer stresses at the surface of the jet and the cone, this results in the suppression of the axisymmetric instability in favor of the jet whipping instability, at high voltage and for highly conductive precursors [31,32].

Figure 2c,d shows the morphology of a composite nanofiber web electrospun at 16 kV applied voltage and 0.5 mL/h feed rate, which are in-between conditions reported for pure and composite PVA precursors [26,34]. While the nanofiber surface roughness is reduced with protruded structures falling below 100 nm, a decrease in the fiber diameter distribution homogeneity is also discerned, constituted of 3 narrow sub-distributions centered around 196 ± 54, 613 ± 87, 1042 ± 98 nm, resulting in a statistical average diameter of 617 ± 339 nm. The widening of the fiber diameter distribution can potentially stem from a jet splitting instability, or also from the variation of the pulled solution volume in successive cycles at the needle tip from an inadequate ratio between solution delivery and solution pulling electric force, thus resulting in several classes of fiber diameters [35,36].

Finally, Figure 2e,f presents a nanofiber web electrospun in the lower range of the reported conditions for pure PVA electrospinning, at 12 kV applied voltage and 0.6 mL/h solution feed rate, with the nanofibers showing a smooth surface at the micrometer scale [34,37]. The average fiber diameter was calculated as 410 ± 59 nm.

**Figure 3:**
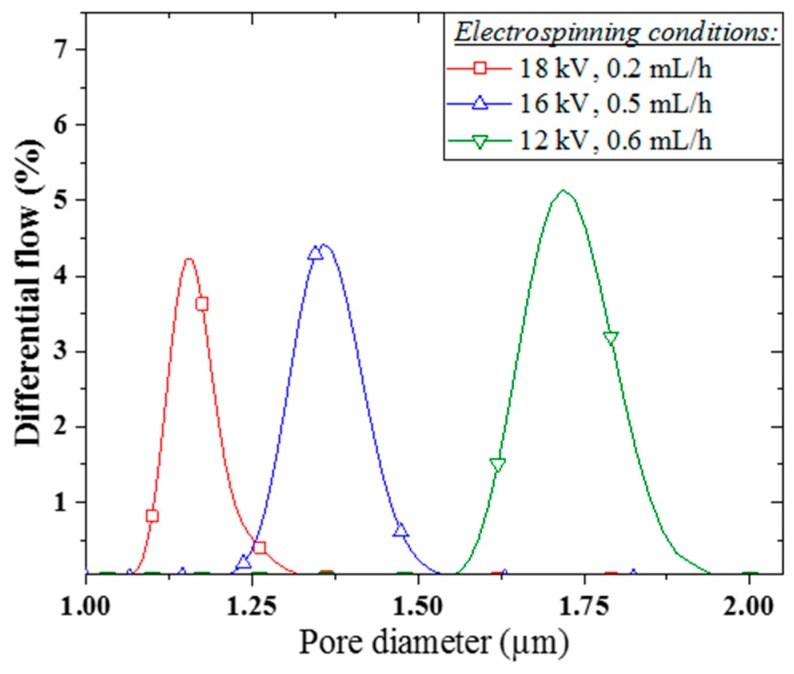
PVA/copper membrane pore size distribution in function of nanofiber electrospinning conditions.

The PVA/copper membranes demonstrated a relatively narrow pore size distribution, typical from an electrospun structure, as presented in Figure 3 [35]. The average membrane pore size was determined to increase from 1.15 to 1.35 and 1.81 μm, with an also increasing full width at half maximum (FWHM) of 110, 128, and 140 nm, for decreasing voltage electrospinning conditions of 18, 16, and 12 kV respectively.

**Figure 4:**
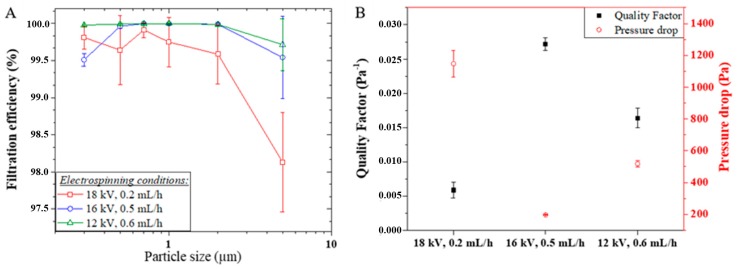
(**a**) Air filtration efficiency performance against 0.3, 0.5, 0.7, 1, 2, and 5 μm KCl crystals. The face velocity applied was 5 cm/s. (**b**) Corresponding parameters of pressure drop and quality factor.

Figure 4 presents the filtration efficiency of the PVA/copper membranes in the filtration of KCl crystals of a size ranging from 0.3 to 5.0 µm, at a face velocity of 5 cm/s, along with a comparison between the membranes quality factor and pressure drop. All three membranes offered good separation performance over 99.5% in average for the [0.3, 2.0] μm particle size range, making them of comparable efficiency with the standard EPA or sub-HEPA filter ISO 25 E, according to the European norm ISO 29463-1 (2011), which categorizes FE regardless of test conditions [38]. The efficiency of the PVA/copper membranes in the removal of PM_5_ was found over 98% in average, thus comparable in efficiency with the standard EPA filter ISO 15 E [38]. The filtration performance given by the PVA-Copper nanofiber membranes is in agreement with other nanofiber air-filtration membranes investigated as transparent air filters, showing a filtration efficiency over 98.5% for PM_2.5_ [39]. For instance, poly(acrylonitrile) PAN nanofiber membranes of 858 nm average fiber diameter showed a filtration efficiency between 96.6 and 99.7% for the (0.3, 5.0) μm particle size range [27].

The pressure drop and the quality factor for the 0.3 μm particle capture (QF_0.3_) accompanying the filtration performance of the PVA/copper membranes was found to be 1237, 196, 518 Pa and 0.006, 0.027, and 0.016 Pa^−1^ respectively with decreasing electrospinning voltage conditions. In comparison, the 858 nm PAN nanofibers reported in [27] demonstrated 96.6% efficiency for 0.3 μm particles in the same conditions of face velocity, with a pressure drop around 130 Pa and a QF_0.3_ of 0.024 Pa^−1^, which is also in the order of performance of the commercial glass fiber filters [27].

The PVA/copper membrane electrospun at 12 kV showed the highest filtration efficiencies (FE) among the three membranes with the FE_0.5–2_ above 99.99%, while FE_0.3_ and FE_5_ were found to be 99.98 and 99.71% respectively. The membrane electrospun at 16 kV followed a similar performance pattern with a decreased efficiency for PM_0.3_ and PM_5_, however accentuated compared to the membrane spun in weaker electric field, with FE_0.3_ and FE_5_ to be 99.51 and 99.54% respectively, while FE_0.5–2_ sits above 99.96%. The membrane electrospun at 18 kV may show a similar behavior when considering the larger error margins, with an accentuated decrease for PM_5_ with a FE_5_ of 98.13% while FE_0.3–2_ is above 99.59%. With nanofiber media and KCl crystals are below 5.0 µm, inertial impaction and interception are the suggested mechanisms of air filtration [5,6,8]. As in nanofiber media less air molecules compared to the larger fibers in similar conditions of membrane pressure drop and fiber porosity impact the fiber surface, thus the air molecules are more able to preserve their initial flow velocity, known as slip-flow [6,8]. This slip-flow phenomenon implies that more KCl crystals will travel near to the fiber, thus increasing interception and inertial impaction [8]. The lower FE shown by the membrane electrospun at 18 kV can be correlated to the too high pressure drop of 1237 Pa, thus affecting the result accuracy, though a high resistance of airflow would point toward a major impaction PM capture phenomenon. While the three membranes were considered to be of similar thickness, the membrane electrospun at 18 kV possessed a thickness 9 and 15 μm greater than the membranes electrospun at 16 and 12 kV respectively.

The membrane average pore size in the considered (1.1, 1.8) μm range can be seen to have a limited impact on the membrane performance from the similarities observed in the FE of the membranes electrospun at 12 and 16 kV respectively, which showed a pore size of 1.35 and 1.81 μm. As a different fiber diameter will affect the membrane separation performance as it implies a different contact surface area and a different length for the PM to cover in order to slip around the fibers, a significant variation in separation was expected across the three membranes [6]. However, both membranes electrospun at 12 and 18 kV with a respective average diameter of 410 and 318 nm showed up to 0.5% of difference in FE_0.5–2_, while a FE_0.5–2_ difference below 0.1% was observed between the membranes electrospun at 12 and 16 kV with a respective diameter of 410 and 617 nm. Considering a slip-flow fiber-particle interaction, a non-homogeneous fiber diameter distribution across the membrane will impact the balance of the forces exerted on the particle [40]. While the difference in fiber contact surface area may influence the forces of adhesion and friction exerted on the particle, noting that particle diameter can be smaller than the fiber diameter for electrospun webs, the potential difference in proximity between the fibers from a diameter variation can also affect the shear flow hence the drag and lift force [40]. However, the impact of the fiber diameter distribution homogeneity was not found determinant, for the membrane electrospun at 18 kV showed the poorest FE with a fiber diameter distribution spanning between 220 and 570 nm, while higher FE were obtained with the 16 kV electrospun membrane, with a wider distribution divided in three classes in the vicinity of 200, 600, and 1050 nm, and with the 12 kV electrospun membrane, with a narrower fiber diameter distribution of three classes around 300, 400, and 460 nm. Fiber surface roughness here plays a major role in the difference of performance between the nanofiber membranes. Indeed, a localized surface texture on the nanofiber can significantly affect the balance between friction and adhesion forces exerted on the KCl crystal at the fiber surface [41,42]. In this study, the observed FE were found improved with decreasing voltage conditions of electrospinning, translating a decreasing nanoscale roughness respectively from above to below 100 nm, homogeneously distributed on the fiber surface, and smooth for the 12 kV electrospun nanofibers. This trend is not always followed in the literature, with reports of hierarchically structured nanoparticle-on-nanofiber composite membranes fabricated for high efficiency air filtration [43].

**Figure 5:**
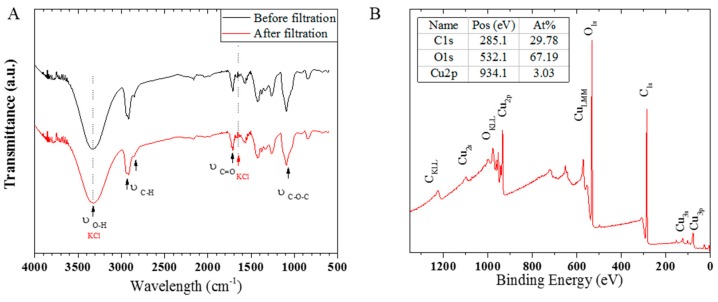
(**a**) Fourier transform infrared (FTIR) spectra obtained at the surface of a poly(vinyl alcohol) (PVA)/copper membrane before and after air filtration test. (**b**) X-ray photospectroscopy (XPS) survey spectra at the surface of a PVA/copper membrane before air-filtration.

A FTIR spectra performed at the surface of a PVA/copper nanofiber web before and after air filtration test is presented in Figure 5a, showing that no absorption of KCl crystal occurred on the PVA/copper nanofibers during air filtration, from the peaks accurate superposition [44]. An XPS survey analysis at the nanofiber surface, presented in Figure 5b, revealed a relative concentration of 3 at % of copper bonds, while oxygen and carbon bonds respectively accounted for 67 and 30 at %. Considering the copper oxidation from air exposure, the organic composition of the nanofibers, and the adventitious carbon at their surface, the found percentage of surface copper bonds was deemed in agreement with a polymer-metal acetate crosslinking previously proposed in Equations (1,2). Furthermore, the presence of copper at the composite nanofibers surface attest of the anti-bacterial potential of PVA/copper nanofiber air-microfilters. The distinctive shape of the Cu_LMM_ peak and the noticeable presence of the Cu2p_1/2_ and Cu2p_3/2_ satellite peaks on the survey spectra indicate the predominant presence of a Cu (II) species, which is in adequation with CuO from surface oxidation under air exposure [45]. As the average nanofiber diameter was not found to significantly impact the filtration performance of the PVA/copper membranes, a decrease in the nanofiber diameter can thus be a potential way to increase the presence of copper at the nanofiber surface from an increased contact surface area.

### 3.2. Anti-Bacterial PVA/Copper Nanofibers

**Figure 6:**
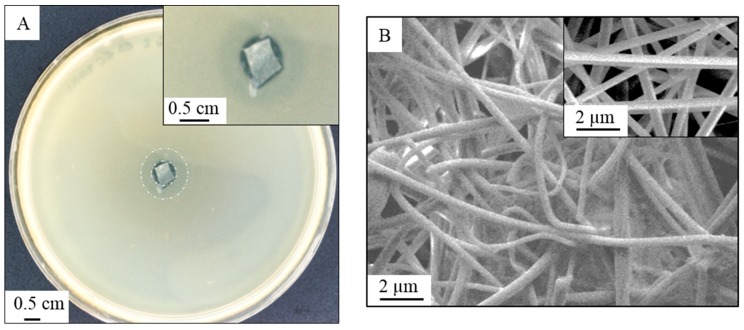
(**a**) Microscope image of an electrospun PVA/copper nanofiber web showing the inhibition zone against *E. coli* after 24 h, following a qualitative test procedure adapted from standard ISO 201645. Inhibition zone measured of 1.2 mm. (**b**) SEM micrograph of the nanofiber web after anti-bacterial test. (insert) SEM micrograph of the reference nanofiber web.

The antibacterial effect of a PVA/Copper nanofiber membrane via assessment of the inhibition zone is shown in Figure 6a according to an adapted protocol from standard ISO 20645 (2004) [28]. The nanofiber web shows an inhibition zone of 1.2 mm after 24 h of incubation from an inoculation of 1–5 × 10^8^ CFU/mL. According to the standard protocol, an inhibition zone over 1 mm demonstrates a good anti-bacterial effect [28]. A SEM micrograph of the membrane after test is presented in Figure 6b showing a moisture resistance behavior of the nanofibers with no degradation or swelling, and limited fouling of the membrane, with no live bacteria present on the fiber web. The nanofibers do not exhibit a smooth structure anymore, with the presence of copper-based microcrystals on their surface, with a homogeneous distribution all over the fiber web.

In the case of metal nanoparticle biocides, reports in the literature suggest that electrostatic interaction between the positively charged nanoparticle and negatively charged bacteria are crucial for the material biocide potential [9]. Here it is believed to be the case with the reaction between the nanofiber and the bacteria entailing the reduction of the copper present on the nanofiber surface. In the case of *Escherichia coli*, chromosomally encoded copper detoxification systems exist to tolerate and regulate the presence of copper within the bacteria [25]. Indeed, the excess Cu (I) is transported from the cytoplasm to the periplasm by the CopA ATPases, which resides in the cytoplasm membrane [25,46,47]. In the periplasm, the copper level is regulated by the copper efflux system CusCFBA, while the copper redox state is controlled by the enzyme CueO, multicopper oxidase [25,46]. Contact killing is here suggested to be the anti-microbial action mechanism of PVA/copper nanofibers, with the disruption of the metabolic processes happening via the deregulation of the three encoding systems defined above, namely *copA*, *cus*, and *cueO*, thus making the bacteria vulnerable to the presence of copper ions. In turn, the proximity of copper ions can generate intracellular superoxide or another reactive oxygen species, following a redox-type reaction between nanofiber and gram negative bacteria [47].

Two circular zones around the fiber sample can be distinguished in Figure 6a, highlighted by two white dashed rings. While the inner circle delimitates the total inhibition zone of 1.2 mm, the outer circle of 3.5 mm radius testifies the PVA-crosslinked copper rate of diffusion. Copper dissolution from the PVA matrix is suggested to happen by reduction or chelative dissolution from the contact with anionic species in *E. coli*, as it is in the reduced state Cu (I) that copper will diffuse in the cytoplasmic membrane, disturbing copper homeostasis [25,46]. While the nature of the copper species involved will influence the metal dissolution rate, substitution and crystal size and order will also have a significant impact [48]. In that sense, it can be argued that the rate of copper dissolution from sol–gel precipitates across PVA chains is more controlled compared to a dissolution from copper metal or oxide crystals.

## 4. Conclusions

PVA crosslinked with copper acetate in a polymer to metal ratio of 60:40 wt % composite nanofiber webs have been successfully fabricated by electrospinning. A weaker applied voltage, compared to those reported in literature was found beneficial in the electrospinning of the composite precursor, reaching smoother nanofiber surfaces and narrower nanofiber diameter distributions. The lower energetic demand was suggested to be associated with the rate of crosslinking between PVA and copper ions, resulting in a molecularly ordered conductive precursor. The PVA/copper nanofiber membranes offered a high air filtration performance with over 99.99% FE for PM_0.5–2_, which covers the size range of bacteria such as gram-negative *Legionella pneumophila*. Nanofiber surface roughness, and in a lower measure nanofiber average diameter and diameter distribution, were found to have an impact on the filtration performance from their impact on the inertial impaction and interception PM capture phenomenon. Furthermore, membrane thickness also played a crucial role with an increase of 15 µm in membrane thickness largely contributed to an increase in pressure drop of 972 Pa. The crosslinked PVA/copper nanofibers demonstrated a good anti-bacterial activity against gram-negative *Escherichia coli*, while also limiting copper diffusion. The nanofibers also showcased a moisture resistance behavior, attributed to the crosslinking that prevented the hydrolysis of PVA chains. Hence PVA/copper electrospun membranes are a promising green material solution for means of air sanitization such as anti-microbial portable air-microfilters.

## Figures and Tables

**Table 1:** Electrospinning parameters used for the materials synthesis.

Electric Field (EF)		Feed Volume (FV)	
Absolute Voltage (kV)	Needle Tip-Collector Distance (cm)	Needle Gauge	Feed Rate (mL/h)
18	20	18 G–ID 0.84 mm	0.2
16	18	21 G–ID 0.51 mm	0.5
12	18	21 G–ID 0.51 mm	0.6

## References

[1] Qian J., Hospodsky D., Yamamoto N., Nazaroff W.W., Peccia J. (2012). Size-resolved emission rates of airborne bacteria and fungi in an occupied classroom. Indoor Air.

[2] Luksamijarulkul P., Pipitsangjan S. (2015). Microbial Air Quality and Bacterial Surface Contamination in Ambulances During Patient Services. Oman Med. J..

[3] Haas K.-H., Luther W., Zweck A. (2013). Industrial Relevant Production Processes for Nanomaterials and Nanostructures. Safety Aspects of Engineered Nanomaterials.

[4] Min K., Kim S., Kim S. (2018). Silk protein nanofibers for highly efficient, eco-friendly, optically translucent, and multifunctional air filters. Sci. Rep..

[5] Podgórski A., Bałazy A., Gradoń L. (2006). Application of nanofibers to improve the filtration efficiency of the most penetrating aerosol particles in fibrous filters. Chem. Eng. Sci..

[6] Balamurugan R., Sundarrajan S., Ramakrishna S. (2011). Recent Trends in Nanofibrous Membranes and Their Suitability for Air and Water Filtrations. Membranes.

[7] Ramakrishna S., Fujihara K., Teo W.-E., Yong T., Ma Z., Ramaseshan R. (2006). Electrospun nanofibers: Solving global issues. Mater. Today.

[8] Electrospintech Air Filtration with Electrospun Nanofibers. http://electrospintech.com/airfilter.html#.W1WbGtIzY2w.

[9] Lala N.L., Ramaseshan R., Bojun L., Sundarrajan S., Barhate R.S., Ramakrishna S., Ying-Jun L. (2007). Fabrication of nanofibers with antimicrobial functionality used as filters: Protection against bacterial contaminants. Biotechnol. Bioeng..

[10] Zhu M., Hua D., Pan H., Wang F., Manshian B., Soenen S.J., Xiong R., Huang C. (2018). Green electrospun and crosslinked poly(vinyl alcohol)/poly(acrylic acid) composite membranes for antibacterial effective air filtration. J. Colloid Interface Sci..

[11] Daels N., De Vrieze S., Sampers I., Decostere B., Westbroek P., Dumoulin A., Dejans P., De Clerck K., Van Hulle S. (2011). Potential of a functionalised nanofibre microfiltration membrane as an antibacterial water filter. Desalination.

[12] Kim S.Y. (2012). Survival of Microorganisms on Antimicrobial Filters and the Removal Efficiency of Bioaerosols in an Environmental Chamber. J. Microbiol. Biotechnol..

[13] Tan K., Obendorf S.K. (2007). Fabrication and evaluation of electrospun nanofibrous antimicrobial nylon 6 membranes. J. Membr. Sci..

[14] Zhao F., Chen S., Hu Q., Xue G., Ni Q., Jiang Q., Qiu Y. (2017). Antimicrobial three dimensional woven filters containing silver nanoparticle doped nanofibers in a membrane bioreactor for wastewater treatment. Sep. Purif. Technol..

[15] Dumée L.F., Yi Z., Tardy B., Merenda A., Ligneris E.D., Dagastine R.R., Kong L., Dagastine R. (2017). Silver metal nano-matrixes as high efficiency and versatile catalytic reactors for environmental remediation. Sci. Rep..

[16] Wahid F., Wang H.-S., Lu Y.-S., Zhong C., Chu L.-Q. (2017). Preparation, characterization and antibacterial applications of carboxymethyl chitosan/CuO nanocomposite hydrogels. Int. J. Boil. Macromol..

[17] Cano A.P., Gillado A.V., Montecillo A.D., Herrera M.U. (2018). Copper sulfate-embedded and copper oxide-embedded filter paper and their antimicrobial properties. Mater. Chem. Phys..

[18] Palza H. (2015). Antimicrobial Polymers with Metal Nanoparticles. Int. J. Mol. Sci..

[19] Haider A., Haider S., Kang I.-K. (2015). A comprehensive review summarizing the effect of electrospinning parameters and potential applications of nanofibers in biomedical and biotechnology. Arab. J. Chem..

[20] Hansen N.S., Cho D., Joo Y.L. (2012). Metal Nanofibers with Highly Tunable Electrical and Magnetic Properties via Highly Loaded Water-Based Electrospinning. Small.

[21] Bognitzki M., Becker M., Graeser M., Massa W., Wendorff J.H., Schaper A., Weber D., Beyer A., Gölzhäuser A., Greiner A. (2006). Preparation of Sub-micrometer Copper Fibers via Electrospinning. Adv. Mater..

[22] Barakat N.A.M., Kim B., Kim H.Y. (2009). Production of Smooth and Pure Nickel Metal Nanofibers by the Electrospinning Technique: Nanofibers Possess Splendid Magnetic Properties. J. Phys. Chem. C.

[23] Ke X.B., Zhu H.Y., Gao X.P., Liu J.W., Zheng Z.F. (2007). High-Performance Ceramic Membranes with a Separation Layer of Metal Oxide Nanofibers. Adv. Mater..

[24] Wu H., Hu L., Rowell M.W., Kong D., Cha J.J., McDonough J.R., Zhu J., Yang Y., McGehee M.D., Cui Y. (2010). Electrospun Metal Nanofiber Webs as High-Performance Transparent Electrode. Nano Lett..

[25] Grass G., Rensing C., Solioz M. (2011). Metallic Copper as an Antimicrobial Surface. Appl. Environ. Microbiol..

[26] Khalil A., Hashaikeh R., Jouiad M. (2014). Synthesis and morphology analysis of electrospun copper nanowires. J. Mater. Sci..

[27] Al-Attabi R., Dumée L.F., Kong L., Schütz J.A., Morsi Y. (2018). High Efficiency Poly(acrylonitrile) Electrospun Nanofiber Membranes for Airborne Nanomaterials Filtration. Adv. Eng. Mater..

[28] International Organization for Standardization (2004). ISO 20645:2004-Textile Fabrics—Determination of Antibacterial Activity—Agar Diffusion Plate Test.

[29] He D., Hu B., Yao Q.-F., Wang K., Yu S.-H. (2009). Large-Scale Synthesis of Flexible Free-Standing SERS Substrates with High Sensitivity: Electrospun PVA Nanofibers Embedded with Controlled Alignment of Silver Nanoparticles. ACS Nano.

[30] Wang H., Lu X., Zhao Y., Wang C. (2006). Preparation and characterization of ZnS:Cu/PVA composite nanofibers via electrospinning. Mater. Lett..

[31] Shin Y., Hohman M., Brenner M., Rutledge G. (2001). Experimental characterization of electrospinning: The electrically forced jet and instabilities. Polymer.

[32] Zuo W., Zhu M., Yang W., Yu H., Chen Y., Zhang Y. (2005). Experimental study on relationship between jet instability and formation of beaded fibers during electrospinning. Polym. Eng. Sci..

[33] Kilic A., Oruc F., Demir A. (2008). Effects of Polarity on Electrospinning Process. Text. Res. J..

[34] Zhang C., Yuan X., Wu L., Han Y., Sheng J. (2005). Study on morphology of electrospun poly(vinyl alcohol) mats. Eur. Polym. J..

[35] Reneker D.H., Yarin A.L. (2008). Electrospinning jets and polymer nanofibers. Polymer.

[36] Reneker D.H., Yarin A.L., Fong H., Koombhongse S. (2000). Bending instability of electrically charged liquid jets of polymer solutions in electrospinning. J. Appl. Phys..

[37] Supaphol P., Chuangchote S. (2008). On the electrospinning of poly(vinyl alcohol) nanofiber mats: A revisit. J. Appl. Polym. Sci..

[38] International Organization for Standardization (2011). ISO 29463-1:2011 High-Efficiency Filters and Filter Media for Removing Particles in Air—Part 1: Classification, Performance Testing and Marking. https://www.iso.org/standard/51835.html.

[39] Liu C., Hsu P.-C., Lee H.-W., Ye M., Zheng G., Liu N., Li W., Cui Y. (2015). Transparent air filter for high-efficiency PM2.5 capture. Nat. Commun..

[40] Sambaer W., Zatloukal M., Kimmer D. (2011). 3D modeling of filtration process via polyurethane nanofiber based nonwoven filters prepared by electrospinning process. Chem. Eng. Sci..

[41] Jin C., Normani S.D., Emelko M.B. (2015). Surface Roughness Impacts on Granular Media Filtration at Favorable Deposition Conditions: Experiments and Modeling. Environ. Sci. Technol..

[42] Fenglei Z., Zhang S., Liu H., Fong H., Yin X., Yu J., Ding B. (2017). Free-Standing Polyurethane Nanofiber/Nets Air Filters for Effective PM Capture. Small.

[43] Su J., Yang G., Cheng C., Huang C., Xu H., Ke Q. (2017). Hierarchically structured TiO_2_/PAN nanofibrous membranes for high-efficiency air filtration and toluene degradation. J. Colloid Interface Sci..

[44] Liu R., Lu H., Wang L., Tian M., Sun W. (2019). Utilization of Ammonium Chloride as a Novel Selective Depressant in Reverse Flotation of Potassium Chloride. Minerals.

[45] Biesinger M.C. (2017). Advanced analysis of copper X-ray photoelectron spectra. Surf. Interface Anal..

[46] Rensing C., Grass G. (2003). Escherichia coli mechanisms of copper homeostasis in a changing environment. FEMS Microbiol. Rev..

[47] Outten F.W., Huffman D.L., Hale J.A., O’Halloran T.V. (2001). The Independent cue and cusSystems Confer Copper Tolerance during Aerobic and Anaerobic Growth in Escherichia coli. J. Biol. Chem..

[48] Schwertmann U. (1991). Solubility and dissolution of iron oxides. Plant Soil.

